# Draft Genome Sequence of *Lactobacillus fermentum* Lf1, an Indian Isolate of Human Gut Origin

**DOI:** 10.1128/genomeA.00883-13

**Published:** 2013-11-14

**Authors:** Sunita Grover, Vineet K. Sharma, Rashmi H. Mallapa, Virender K. Batish

**Affiliations:** Molecular Biology Unit, Department of Dairy Microbiology, National Dairy Research Institute, Karnal, Haryana, Indiaa; Metagenomics and Systems Biology Laboratory, Indian Institute of Science Education and Research (IISER), Bhopal, Indiab

## Abstract

*Lactobacillus fermentum* is a normal inhabitant of the human gastrointestinal tract. Here, we report the draft genome sequence of an Indian isolate of the probiotic strain *L. fermentum* Lf1, isolated from the human gut.

## GENOME ANNOUNCEMENT

*Lactobacillus fermentum*, a heterofermentative species of the genus *Lactobacillus*, is a normal inhabitant of the human gastrointestinal tract and possesses probiotic attributes other than its commercial uses in food and feed fermentation. The complete whole-genome sequences of strains *L. fermentum* IFO 3956 and *L. fermentum* CECT 5716, a probiotic strain from human milk, have been determined (1, 2). Recently, Maldonado et al. (3) reported that strain CECT 5716 may be useful for the prevention of community-acquired gastrointestinal and upper respiratory infections. *L. fermentum* strains E-3 and E-18 have also been reported to possess strong antioxidative properties (4).

*L. fermentum* Lf1, an indigenous isolate from the human gut, was identified using 16S rRNA (accession no. KC509914) and *rpoA* (accession no. KC509912). It has been deposited in the International Depository Budapest Treaty at the Microbial Type Culture Collection (MTCC) as strain MTCC 5689. Lf1 possesses probiotic attributes, *viz*. in acid and bile tolerance and autoaggregation ability, and it inhibits gastrointestinal pathogens. It has also been found to upregulate the transcription factor Nrf2 and inhibits lipid peroxidation (R. Chauhan, V. K. Batish, and S. Grover, unpublished data). The functional efficacy of Lf1 in terms of its anti-inflammatory and antioxidative properties was demonstrated in a dextran sodium sulfate (DSS)-induced colitis mouse model. The disease activity index (DAI) and histological scores of the colitis mouse group fed with Lf1 improved significantly compared to those in colitis mice, and the Lf1-fed group prevented lipid peroxidation. Since Lf1 might be an ideal probiotic, its genomic sequence was deciphered.

Genome sequencing was performed using Illumina Solexa genome analyzer (HiScanSQ genome analyzer; Illumina, Inc.). A total of 7,665,410 high-quality reads were obtained after filtering with a Phred score of <20, and these were used for alignment with *L. fermentum* IFO 3956 (accession no. NC_010610.1) as the reference genome. Of the total reads, 83.50% were aligned with the reference genome, with 86.90% genome coverage and total gap length of 0.27 Mb. A genome comparison of Lf1 with that of *L. fermentum* IFO 3956 revealed 9,210 single-nucleotide polymorphisms (SNPs), which were mapped to 653 genes.

The draft genome of Lf1 was assembled into a single circular chromosome of 2,098,685 bp using the reference *L. fermentum* IFO 3956 sequence using the Burrows-Wheeler Aligner (BWA) (5) to insert Ns to fill the gaps. The G+C content of Lf1 is 52.5%, and 1,620 predicted protein-coding genes were identified by using Glimmer 3.02 (6), followed by manual curation. A total of 1,233 (76.1%) genes were annotated with known functions using BLASTp against the NCBI nonredundant (NR) and Clusters of Orthologous Groups (COG) databases (7). Three hundred eighty-seven (23.9%) genes were annotated as hypothetical, conserved hypothetical, or unnamed protein products. The Lf1 strain contains five rRNA gene operons (5S, 16S, and 23S rRNAs) and 57 tRNA genes in the genome. A total of 1,389 genes were classified into 20 functional COGs, of which the most abundant classes were replication, recombination, and repair (18%), amino acid transport and metabolism (13%), and transcription (11%).

### Nucleotide sequence accession numbers.

The *L. fermentum* Lf1 whole-genome shotgun project has been deposited at DDBJ/EMBL/GenBank under the accession no. AWXS00000000. The version described in this paper is version AWXS01000000.
